# Weight loss improves β-cell function independently of dietary carbohydrate restriction in people with type 2 diabetes: A 6-week randomized controlled trial

**DOI:** 10.3389/fnut.2022.933118

**Published:** 2022-08-19

**Authors:** Mads N. Thomsen, Mads J. Skytte, Amirsalar Samkani, Arne Astrup, Mogens Fenger, Jan Frystyk, Bolette Hartmann, Jens J. Holst, Thomas M. Larsen, Sten Madsbad, Faidon Magkos, Jens F. Rehfeld, Steen B. Haugaard, Thure Krarup

**Affiliations:** ^1^Department of Endocrinology, Copenhagen University Hospital Bispebjerg, Copenhagen, Denmark; ^2^Healthy Weight Center, Novo Nordisk Foundation, Hellerup, Denmark; ^3^Department of Clinical Biochemistry, Copenhagen University Hospital Hvidovre, Copenhagen, Denmark; ^4^Department of Endocrinology, Odense University Hospital, Odense, Denmark; ^5^Department of Clinical Medicine, Aarhus University, Aarhus, Denmark; ^6^Novo Nordisk Foundation Center for Basic Metabolic Research, Copenhagen, Denmark; ^7^Department of Biomedical Sciences, University of Copenhagen, Copenhagen, Denmark; ^8^Department of Nutrition, Exercise and Sports, University of Copenhagen, Copenhagen, Denmark; ^9^Department of Endocrinology, Copenhagen University Hospital Hvidovre, Copenhagen, Denmark; ^10^Department of Clinical Biochemistry, Rigshospitalet, University of Copenhagen, Copenhagen, Denmark

**Keywords:** β-cell function, carbohydrate restriction, insulin sensitivity, low-grade inflammation, type 2 diabetes, weight loss

## Abstract

**Background:**

Carbohydrate restriction may benefit β-cell function and glucose metabolism in type 2 diabetes (T2D) but also leads to weight loss which in itself is beneficial.

**Methods:**

In order to determine the additional effect of carbohydrate restriction in addition to a fixed body weight loss, we randomly assigned 72 adults with T2D and obesity (mean ± SD HbA_1c_ 7.4 ± 0.7%, BMI 33 ± 5 kg/m^2^) to a carbohydrate-reduced high-protein diet (CRHP; energy percent from carbohydrate/protein/fat: 30/30/40) or an isocaloric conventional diabetes diet (CD; 50/17/33) for 6 weeks. All foods were provided free of charge and total energy intake was tailored individually, so both groups lost 6% of baseline body weight.

**Results:**

Despite significantly greater reductions in HbA_1c_ (mean [95% CI] −1.9 [−3.5, −0.3] mmol/mol) after 6 weeks, the CRHP diet neither improved glucose tolerance, β-cell response to glucose, insulin sensitivity, during a 4-h oral glucose tolerance test, nor basal proinsulin secretion when compared to the CD diet, but increased C-peptide concentration and insulin secretion rate (area under the curve [AUC] and peak) significantly more (~10%, *P* ≤ 0.03 for all). Furthermore, compared with the CD diet, the CRHP diet borderline increased basal glucagon concentration (16 [−0.1, 34]%, *P* = 0.05), but decreased glucagon net AUC (−2.0 [−3.4, −0.6] mmol/L ×240 min, *P* < 0.01), decreased basal triglyceride and total AUC (~20%, *P* < 0.01 for both), and increased gastric inhibitory polypeptide total AUC (14%, *P* = 0.01).

**Conclusion:**

A moderately carbohydrate-restricted diet for 6 weeks decreased HbA_1c_ but did not improve β-cell function or glucose tolerance beyond the effects of weight loss when compared with a conventional diabetes diet in people with T2D.

**Clinical trials registration:**

www.Clinicaltrials.gov, Identifier: NCT02472951.

## Introduction

Type 2 diabetes (T2D) is characterized by hyperglycemia, which occurs when insulin resistance is not adequately compensated by hyperinsulinemia. In fact, despite ongoing insulin resistance, T2D does not manifest until pancreatic β-cells fail to produce sufficient amounts of insulin to maintain normal glucose tolerance (1). At this point, patients have lost over 80% of their β-cell function and the overworked β-cells are believed to be “exhausted” although the exact mechanisms are yet to be identified (2). “Lipotoxicity,” i.e., chronically elevated levels of circulating non-esterified fatty acids (NEFAs), may impair normal insulin secretion (3), but the weight of evidence suggests that “glucotoxicity” is primarily responsible for β-cell function impairment (4). Indeed, short-term amelioration of hyperglycemia by intensive insulin therapy may to some extent improve β-cell responsiveness to glucose and incretin hormones (5, 6). Furthermore, decreasing the β-cell workload, without changing plasma glucose concentrations, by overnight exogenous infusion of somatostatin, enhances first-phase insulin secretion and decreases proinsulin/insulin ratio (7), which is suggestive of more appropriate intracellular insulin processing and less β-cell stress (8). Accordingly, increased plasma proinsulin relative to insulin or C-peptide has been suggested to be a sensitive index of β-cell failure, often found in individuals with T2D in the fasted state but, particularly, when requirements for insulin secretion increase (9).

Lifestyle modification is pivotal in T2D management, with weight loss being the cornerstone (10). In fact, remission of T2D can be achieved after a >15% weight loss in most patients with short-term diabetes (11), partly associated with the recovery of first-phase insulin secretion, traditionally believed to be irreversibly lost in long-lasting T2D (12). Even without significant changes in body weight, however, several meta-analyses have suggested that additional metabolic benefits, in terms of lower concentrations of glycated hemoglobin (HbA_1c_), triglyceride, and high-density lipoprotein cholesterol, are achievable following dietary carbohydrate restriction, at least in the short term (13–15). Accordingly, carbohydrate restriction has been suggested as the first approach in diabetes management (16), and the American Diabetes Association (ADA) recently recognized carbohydrate restriction as a viable dietary strategy to improve glycemic control (17).

Previously, we demonstrated that a carbohydrate-reduced high-protein (CRHP) diet improved glycemic control and lipid metabolism when compared with a conventional diabetes (CD) diet (18), which noteworthy also improved β-cell responsiveness to glucose and proinsulin processing although the participants had a mean T2D duration of 7 years (19). Thus, the loss of β-cell function may be at least partially restored by restricting carbohydrate intake independently of weight loss, even in patients with a longer duration of T2D. Unfortunately, carbohydrate-restricted eucaloric diets readily reduce body weight despite considerable efforts to prevent this from happening (18, 20); hence, the results of carbohydrate restriction beyond weight loss are often difficult to interpret. Accordingly, we aimed to evaluate the effects of matched 6% weight loss, induced by 6 weeks of a hypocaloric CRHP or CD diet, on β-cell function and insulin sensitivity, and pancreatic and gut hormone secretion in people with T2D and overweight or obesity. The current study represents a secondary analysis of a trial for which the primary and secondary outcomes (glycemic control, basal triglyceride, and ectopic fat) have been reported elsewhere (21).

## Materials and methods

### Study design and eligibility criteria

This open-labeled, parallel, randomized clinical trial was conducted at Copenhagen University Hospital Bispebjerg from January 2018 to July 2019 and included people with T2D from the Capital Region of Denmark. A full list of eligibility criteria has been provided previously (21). In brief, individuals with an HbA_1c_ of 6.5–11.0% (48–97 mmol/mol), body mass index (BMI) >25 kg/m^2^, and glucose-lowering therapy restricted to metformin and/or dipeptidyl peptidase 4 (DPP-4) inhibitors, and without critical illness, renal dysfunction (estimated glomerular filtration rate <30 mL/min/1.73 m^2^ or urine albumin/creatinine ratio >300 mg/g), and without treatment with systemic corticosteroids, sulfonylureas, sodium–glucose co-transporter 2 inhibitors or injectable hypoglycemic medications, were eligible for enrolment. The patients consented in writing, after appropriate oral and written information, to participate in the study, which was approved by the Health Ethics Committee of Copenhagen and the Danish Data Protection Agency. The study was registered with clinicaltrials.gov (NCT02472951) and was conducted in accordance with the Declaration of Helsinki.

### Diet intervention and weight loss management

Participants were randomly assigned in a 1:1 ratio to 6 weeks of a fully provided hypocaloric CD or CRHP diet consisting of 50 and 30% of total energy (E%) from carbohydrates, 17 and 30 E% from proteins, and 33 and 40 E% from fats, respectively. Other dietary components varied with the foods included, for instance, the contents of monounsaturated fat (higher in the CRHP diet) and fiber (higher in the CD diet; Supplementary Table 1). Diets were provided free of charge two times weekly and included three main meals with or without two snacks and all daily calories. Alcohol, soft drinks, and any other calorie-containing foods or beverages not provided by the investigational team were not allowed during the study. Participants were instructed to consume all meals, and dietary adherence was evaluated at each diet provision by the use of food records. Meals were prepared in the metabolic kitchen at the Department of Nutrition, Exercise and Sports (NEXS), University of Copenhagen, as seven different daily menus.

The weight loss regimen aimed for a 6% reduction in baseline body weight over the first 5 weeks and stabilization at this new lower body weight during the last week, so that the post-intervention testing was performed in a state of relative energy balance without being confounded by possible acute effects of energy restriction. A fixed weight loss algorithm was applied to each participant, which has been detailed elsewhere (21). To ensure the targeted weight loss, body weight was evaluated two times weekly and, if necessary, adjustments in dietary energy were made by adding or subtracting CRHP or CD food items. Participants were instructed not to change their habitual physical activity during the study, and the International Physical Activity Questionnaire (IPAQ) long form was used at baseline and week 5 to assess adherence. All pharmacotherapy affecting glucose, lipids, or blood pressure was kept constant in the 2 months prior to study commencement and throughout the intervention.

### Oral glucose tolerance test and analyses

An oral glucose tolerance test (OGTT) was performed on weeks 0 and 6 to assess β-cell function, insulin sensitivity, and hormonal responses involved in glucose homeostasis and satiety. Participants were instructed not to participate in any strenuous activities for 48 h prior to testing. In the morning of the testing days, after an overnight 10-h fast, a cannula was inserted in an antecubital vein and two fasting blood samples were drawn (at time points −10 and 0 min). Then, a standardized OGTT solution (75 g glucose dissolved in 300 ml of water) was ingested over 5 min and additional blood samples were drawn at time points 10, 20, 30, 45, 60, 90, 120, 150, 180, 210, and 240 min. Participants remained sedentary in a reclined position throughout the OGTT. Blood was processed accordingly for the separation of plasma or serum and stored at −80°C until analysis.

Plasma was analyzed for glucose, cholecystokinin (CCK), and gastrin at all available time points and glucagon, glucagon-like peptide-1 (GLP-1), and gastric inhibitory polypeptide (GIP) at times 0, 30, 60, 90, 120, 150, 180, and 240 min; whereas serum was used for the measurement of insulin, C-peptide, triglyceride, and NEFAs at every time point (21). Concentrations of total glucagon, GLP-1, and GIP were determined (following extraction from plasma with 70% ethanol) by radioimmunoassay (RIA) using C-terminally directed antisera code nos. 4305, 89390, and 867, respectively (22). Likewise, CCK concentrations were measured by RIA using an antiserum directed at the C-terminal sequence (Ab. no. 92128), which specifically binds all the bioactive forms of CCK without cross-reactivity with any of the homologous gastrin (23). The gastrin concentrations were also measured by RIA, but using an antiserum (Ab. no. 2604), which specifically binds all the bioactive forms of gastrin without cross-reactivity with any of the homologous CCK peptides (24).

Fasting samples (at time point −10 min) were used for measuring C-reactive protein (CRP), tumor necrosis factor (TNF)-α, interleukin (IL)-6, and IL-8 as markers of inflammation, and intact proinsulin (IP) and 32,33 split proinsulin (SP) as indicators of proinsulin processing, as these comprise the major portion of circulating proinsulin-like molecules (25). Serum CRP was measured by enzyme-linked immunosorbent assay (VICTOR Nivo; PerkinElmer, MA, USA), and serum TNF-α, IL-6, and IL-8 by multi-spot immunoassay (V-PLEX; Meso Scale Discovery, MD, USA); all were measured in duplicate where an intra-assay coefficient of variation (CV) of >20% excluded data from analysis. Serum IP and SP were determined in duplicate with a fluorometric immunoassay (Auto-DELFIA; PerkinElmer, MA, USA) with a lower detection limit of 1.25 pmol/L (26). The results were summed to give total proinsulin. Considerable cross-reactivity between these two assays exists which has been accounted for previously (19). The intra-assay CV for duplicate measurements of CRP, TNF-α, IL-6, and IL-8 was 6, 3, 5, and 3%, respectively.

### Outcomes and calculations

Basal concentrations were obtained from samples taken at time point 0 min or as the mean of time points 0 and −10 min when both were available. Responses to the OGTT were evaluated as the area under the curve (AUC), calculated by the trapezoidal method including net AUC (i.e., total AUC minus the area below baseline) where variables suppressed by the OGTT, i.e., triglyceride, NEFAs, and glucagon, took negative values. In addition, we determined the peak/nadir values, defined as the greatest increment/decrement above/below the baseline, as well as the time point when these values were reached.

Prehepatic insulin secretion rates (ISR) were estimated by the deconvolution of C-peptide concentrations using the ISEC software which integrates postprandial peripheral concentrations of C-peptide and individual subject characteristics in a two-compartment model (27). ISR and its relation to plasma glucose were used to evaluate the β-cell responsiveness or sensitivity to glucose in the early phase from 0 to 30 min and expressed as the insulinogenic index (IGI_30_ = [ISR AUC_0−30*min*_]/[glucose AUC_0−30*min*_]). IGI_240_ = [ISR AUC_0−240*min*_]/[glucose AUC_0−240*min*_] was calculated as an index of the full insulin response during the OGTT. B_total_ was calculated as an index of changes in insulin secretion relative to changes in glucose and incretin hormones, represented as the slope of individual regression lines achieved by the cross-correlation of corresponding values of ISR and plasma glucose during the total 240 min of the OGTT.

As a marker of whole-body insulin sensitivity, the composite index (ISI_comp_) was calculated as follows: 10,000/√[basal glucose x basal insulin x $\bar{\text{G}}$ x $\bar{\text{I}}$; where $\bar{\text{G}}$ is mean glucose and $\bar{\text{I}}$ is mean insulin during the 4-h OGTT (28). Included values of glucose and insulin were in units of mg/dL and μU/ml [conversion factor: 1 μU/ml = 6.0 pmol/L (29)], respectively. Given the hyperbolic relationship between insulin sensitivity and β-cell responsiveness to glucose, which has been validated in another study (30), the disposition index, calculated as their product (D_i_ = ISI_comp_ x B_total_), was used to assess β-cell function (31). The metabolic clearance rate of insulin was estimated as the ratio between newly secreted insulin and total serum concentration of insulin throughout the OGTT after adjustment for body weight by using the following formula: MCRi = [ISR AUC_0−240*min*_]/[insulin AUC_0−240*min*_] × body weight (30).

### Sample size, statistics, and randomization

Sample size calculations, together with primary and secondary outcomes, have been reported elsewhere (21). In brief, the study evaluated changes in HbA_1c_ and liver fat as its primary and leading secondary outcomes, respectively, and adequate power was expected with 80 participants enrolled, allowing for 20% attrition. β-cell function, insulin sensitivity, and hormonal responses following the OGTT, presented here, were pre-specified exploratory outcomes and interpreted as such. Accordingly, no adjustments were made for multiplicity in data analysis, and the two-sided statistical tests were considered significant when *P* < 0.05.

Summary statistics included only completing participants. All data—except for net AUCs and time to peak/nadir—were log-transformed because of distribution skewness. Therefore, unless otherwise stated, baseline data are shown as geometric means (95% CI), and changes from baseline are shown as a percentage. Statistical inference was accomplished using all available data in a constrained linear mixed model with inherent baseline adjustment, and the treatment effect was evaluated as the marginal mean (95% CI) of CRHP vs. CD diet. The model used an unstructured pattern of covariance to account for repeated measurements. Missing data were assumed to be missing at random and implicitly handled by maximum likelihood estimation. Model assumptions were assessed from residual diagnostics, and skewed variables were handled by log-transformation prior to analysis, in which case differences between diets are given in percentage. To account for possible confounders, a secondary model was adjusted for the covariates sex, age, duration of T2D, BMI, and therapy with metformin and DPP-4 inhibitors.

An unrelated study nurse was responsible for the randomization which upon the allocation of the participants was unblinded for the investigators; whereas participants were kept blind until the first meal provision. Randomization was performed in blocks of random size through a generated randomization list which was conducted in R (Version 3.6.0, R, Boston, MA, USA) together with all statistical analyses and graphics.

## Results

### Participants and baseline characteristics

From a total of 338 telephone pre-screenings and 102 screening visits, 72 participants were enrolled in the present study of whom 67 completed all visits; three withdrew consent during the intervention, and two before the first visit but after randomization. Reasons for withdrawal have been described elsewhere (21) but were not related to trial outcomes or any study-related adverse events. Because of the COVID-19 pandemic lockdown, fewer than the expected 80 participants were enrolled but power was sufficient due to lower than anticipated attrition rates (CD 8.3%, CRHP 5.6%). Randomization was successful and, apart from uneven distribution of sex and use of DPP-4 inhibitors, the baseline characteristics were well balanced between groups (Table 1). Importantly, the results were robust when adjusting for baseline differences in sex, age, duration of T2D, BMI, and use of metformin and DPP-4 inhibitors.

**Table 1:** Baseline characteristics of participants with T2D.

**Characteristic**	**CD diet**	**CRHP diet**
Participants/white ethnicity, *n*	33/33	34/34
Male/female sex, *n*	15/18	20/14
Age, years	67.0 (±8.8)	66.4 (±6.9)
Duration of T2D, years	7.7 (2.8, 10.1)	8.5 (3.5, 11.9)
HbA_1c_, %	7.40 (±0.70)	7.42 (±0.77)
HbA_1c_, mmol/mol	57.4 (±7.7)	57.6 (±8.4)
Body mass index, kg/m^2^	33.2 (±5.1)	33.6 (±4.6)
Body weight, kg	97.5 (±25.4)	98.0 (±14.2)
Estimated daily TEE, kcal	2,600 (±632)	2,652 (±364)
Medication use, *n* (%)
No glucose-lowering therapy	12 (36)	8 (24)
Glucose-lowering therapy	21 (64)	26 (76)
1 hypo-glycemic agent	18 (54)	16 (47)
2 hypo-glycemic agent	3 (9)	10 (29)
Metformin	21 (64)	25 (74)
DPP-4 inhibitors	3 (9)	11 (32)
Lipid-lowering therapy	23 (70)	26 (76)
Anti-hypertensive therapy	26 (79)	29 (85)

*Data are presented as means (±SD), medians (25th, 75th percentiles), or frequencies.*

*CD, conventional diabetes; CRHP, carbohydrate-reduced high-protein; DPP-4, dipeptidyl peptidase 4; T2D, type 2 diabetes; TEE, total energy expenditure*.

### Weight loss

Weight loss in the two diet groups was well-matched (CHRP vs. CD: 0.1 [−0.6, 0.7] kg, *P* = 0.83) with a 5.8 kg mean decrease in both groups. No differences in dietary energy restriction (4 [−149, 158] kcal/day, *P* = 0.95) or physical activity level (0 [−27, 37]%, *P* = 0.99) were found. These outcomes have been published in detail elsewhere (21).

### Glucose and lipid metabolism

Overall, glycemic control was improved by the CRHP diet after 6 weeks compared with the CD diet as HbA_1c_ decreased by 1.9 [−3.5, −0.3] mmol/mol and diurnal mean glucose by −0.8 (−1.2, −0.4) mmol/L; both reported elsewhere (21).

**Figure 1:**
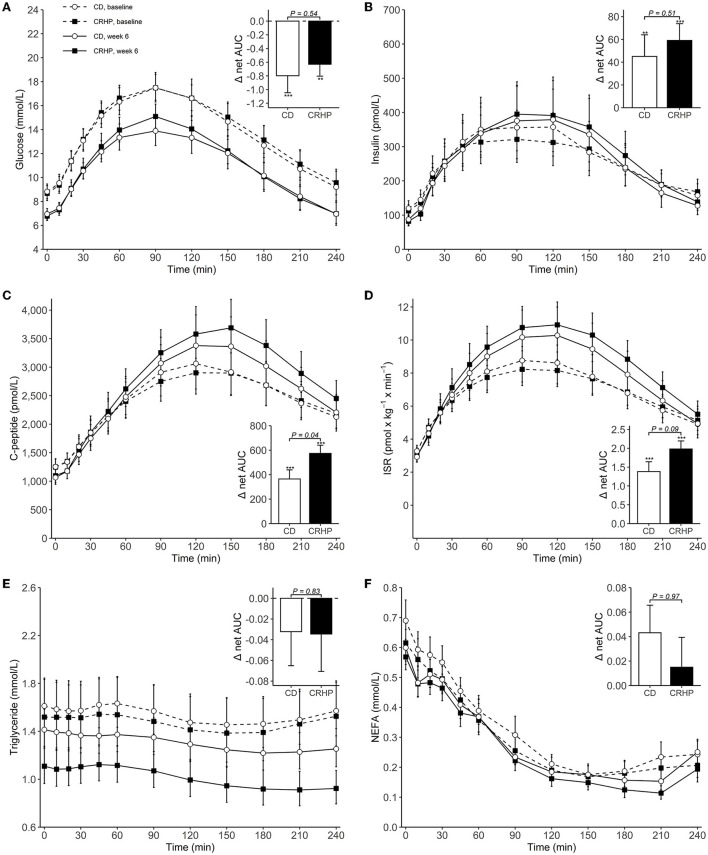
Concentrations of plasma glucose **(A)**, serum insulin **(B)**, serum C-peptide **(C)**, insulin secretion rate (ISR) **(D)**, serum triglyceride **(E)**, and serum non-esterified fatty acids (NEFAs) **(F)** during an OGTT at baseline and after 6 weeks of a CD or CRHP diet. Inserted plots represent net AUC in the units stated x 240 min. Data are presented as mean (±SEM) following log-transformation, except for net AUCs; **P < 0.01; ***P < 0.001.

Basal concentrations of glucose, insulin, C-peptide, and ISR did not differ between diets and were all reduced after the 6-week intervention (Table 2). When compared with baseline, weight loss with both diets increased total AUC, net AUC, peak, and time to peak of insulin, C-peptide, and ISR; and decreased total AUC, net AUC, and peak of glucose (Table 2; Figure 1). After 6 weeks, the CRHP diet increased total AUC (9 [2, 18]%) and net AUC (203 [11, 394] pmol/L ×240 min) of C-peptide and ISR response (10 [1, 19]%) (*P* < 0.05 for all) to a significantly greater extent compared with the CD diet, but no differences in glucose response were found (Table 2; Figure 1). Peak values of C-peptide and ISR also increased to a significantly greater extent by the CRHP than the CD diet (Supplementary Table 2).

**Table 2:** Basal concentrations and responses to an oral glucose tolerance test of glucose, insulin, C-peptide, triglyceride, NEFAs, and insulin secretion at baseline and after matched ~6% weight loss by a CD or a CRHP diet in individuals with T2D and overweight or obesity.

	**CD diet**, ***n*** = **33**		**CRHP diet**, ***n*** = **34**		**Between diets**	
	**Baseline**	**Change^a^**	**Baseline**	**Change^a^**	**Difference^b^**	***P* value**
Plasma glucose						
Basal glucose, mmol/L	8.8 (8.2, 9.5)	−21 (−26, −16)^‡^	8.7 (8.1, 9.3)	−22 (−26, −17)^‡^	−2 (−8, 5)	0.63
AUC, mmol/L x 240 min	13.9 (12.8, 15.1)	−19 (−24, −15)^‡^	14.1 (13.3, 15.0)	−18 (−23, −14)^‡^	1 (−5, 9)	0.70
Serum insulin
Basal insulin, pmol/L	125 (106, 148)	−26 (−36, −16)^‡^	117 (101, 135)	−27 (−35, −18)^‡^	−0.4 (−16, 18)	0.96
AUC, pmol/L x 240 min	281 (223, 355)	2 (−7, 11)	266 (215, 328)	13 (4, 23)^†^	11 (−0.5, 24)	0.06
Serum C-peptide
Basal C-peptide, pmol/L	1,252 (1,125, 1,394)	−15 (−22, −8)^‡^	1,249 (1,126, 1,386)	−12 (−18, −6)^‡^	3 (−6, 14)	0.50
AUC, pmol/L x 240 min	2,501 (2,183, 2,865)	8 (1, 14)^†^	2,464 (2,171, 2,797)	18 (11, 25)^‡^	9 (2, 18)	0.02
Insulin secretion rate						
Basal ISR, pmol x kg^−1^ x min^−1^	3.3 (3.0, 3.6)	−11 (−17, −4)^†^	3.2 (3.0, 3.5)	−8 (−13, −2)^*^	3 (−6, 13)	0.50
AUC, pmol x kg^−1^ x 240	7.2 (6.2, 8.2)	14 (7, 22)^‡^	7.0 (6.2, 7.8)	26 (18, 34)^‡^	10 (1, 19)	0.03
Serum triglyceride						
Basal triglyceride, mmol/L	1.7 (1.5, 1.9)	−13 (−24, −0.7)^*^	1.6 (1.3, 1.9)	−27 (−37, −15)^‡^	−19 (−30, −6)	<0.01
AUC, mmol/L x 240 min	1.5 (1.3, 1.8)	−15 (−25, −5)^†^	1.5 (1.2, 1.8)	−31 (−40, −21)^‡^	−21 (−31, −9)	<0.01
Serum NEFA
Basal NEFA, mmol/L	0.71 (0.64, 0.78)	−11 (−17, −4)	0.61 (0.55, 0.68)	−5 (−14, 6)	−1 (−11, 10)	0.85
AUC, mmol/L x 240 min	0.32 (0.29, 0.36)	−14 (−18, −9)^†^	0.29 (0.26, 0.33)	−13 (−23, −1)^‡^	−4 (−14, 7)	0.46

*Data are presented as mean (95% CI) following log-transformation. Between-diet differences are estimated marginal means (CRHP vs. CD) derived from constrained linear mixed models with inherent baseline adjustment using all available data.*

a *Relative change (%) from baseline.*

b *Relative difference (%) between diets.*

* *P <0.05,*

† *P <0.01, and*

‡ *P <0.001 vs. baseline.*

*CD, conventional diabetes; CRHP, carbohydrate-reduced high-protein; ISR, insulin secretion rate; NEFAs, non-esterified fatty acids*.

Triglyceride and NEFA total AUCs and nadirs were reduced with both diets when compared with baseline. The CRHP diet reduced basal triglyceride (−19 [−30, −6]%) and triglyceride total AUC and nadir (−21 [−31, −9]% and −25 [−35, −13]%, respectively) (*P* < 0.01 for all) and tended to also suppress NEFA nadir (−20 [−36, −0.1]%, *P* = 0.05) to a greater extent than the CD diet.

### Insulin sensitivity and β-cell function

**Figure 2:**
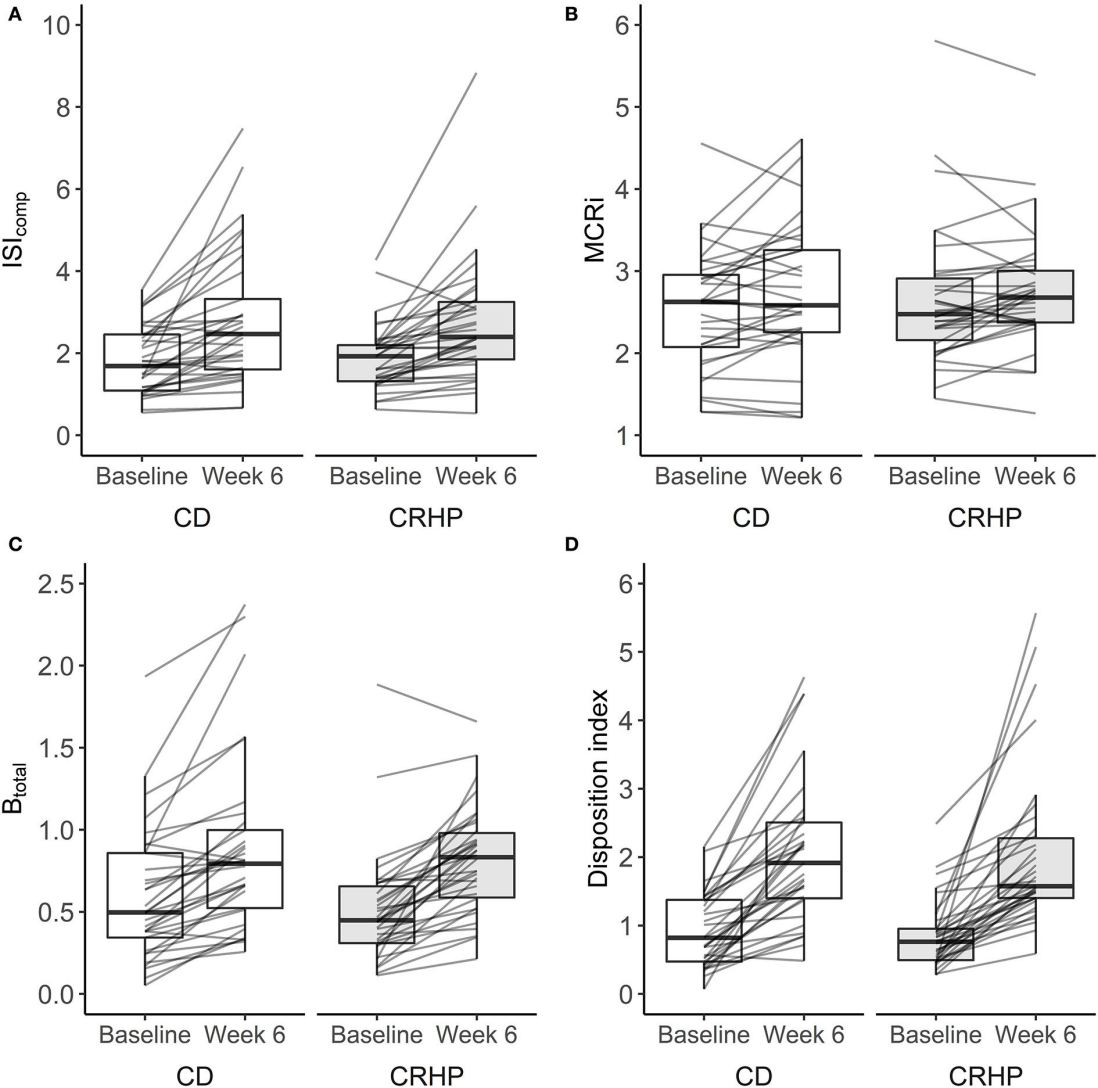
Changes in the composite index (ISI_comp_) **(A)**, metabolic clearance rate of insulin (MCRi) **(B)**, β-cell responsiveness to glucose (B_total_) **(C)**, and disposition index (D_i_) **(D)** derived from an OGTT at baseline and after 6 weeks of a CD or CRHP diet. Data are presented as median (25th, 75th) with individual changes. No differences between diets were evident from linear mixed model analysis: *P* = 0.47, *P* = 0.77, *P* = 0.65, and *P* = 0.99 for **(A–D)**, respectively. Estimates are in units: L^2^ x mg^−1^ × μU^−1^ ×10^−4^ (ISI_comp_), L × min^−1^ (MCRi), L x kg^−1^ × min^−1^ ×10^−9^ (B_total_), and L^3^ × g^−2^ x min^−1^ × μU^−1^ ×10^−1^ (D_i_).

Weight loss induced by either diet significantly increased ISI_comp_ by approximately 42% and indices of β-cell responsiveness (B_total_, IGI_30_, and IGI_240_) and function by 24–68% and 138%, respectively (*P* < 0.001 for all). Improvements were seen in most participants: 88% for ISI_comp_, 93% for B_total_, and 99% for D_i_ (Figure 2). The CRHP diet did not improve measures of insulin sensitivity, β-cell responsiveness to glucose, or β-cell function when compared with the CD diet (Supplementary Table 3). No difference was found for MCRi between diets (Figure 2), and only small changes were observed when compared with baseline.

### Glucagon and gut hormones

**Figure 3:**
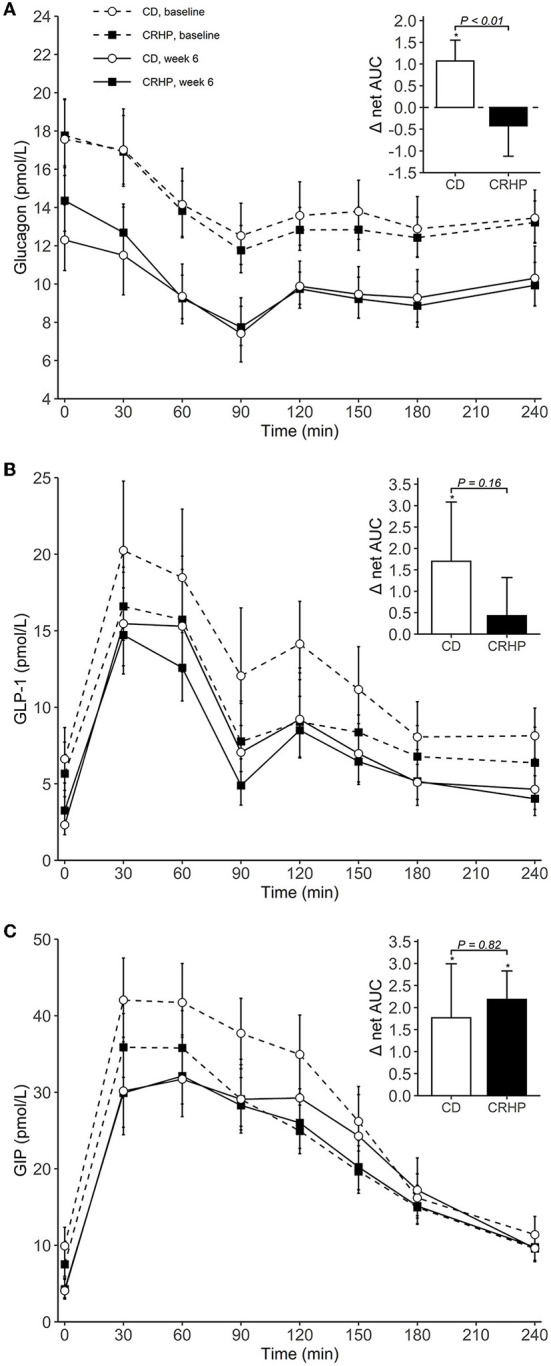
Plasma concentrations of glucagon **(A)**, GLP-1 **(B)**, and GIP **(C)** during an OGTT at baseline and after 6 weeks of a CD or CRHP diet. Inserted plots represent net AUC in the units stated x 240 min. Data are presented as mean (±SEM) following log-transformation, except for net AUCs; *P < 0.05.

**Figure 4:**
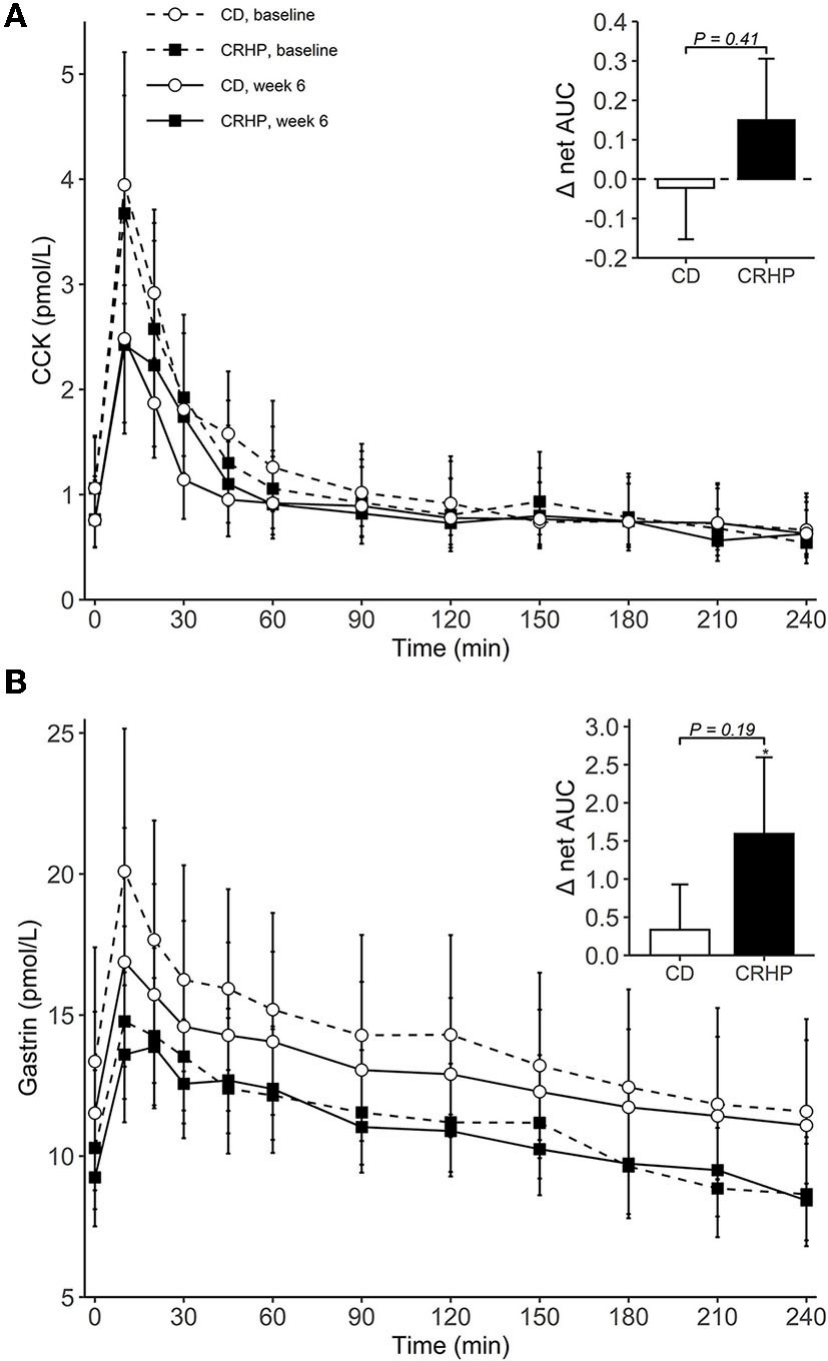
Plasma concentrations of CCK **(A)** and gastrin **(B)** during an OGTT at baseline and after 6 weeks of a CD or CRHP diet. Inserted plots represent net AUC in the units stated x 240 min. Data are presented as mean (±SEM) following log-transformation, except for net AUCs; *P < 0.05.

Changes in basal concentrations of GLP-1, GIP, CCK, and gastrin after 6 weeks were similar between diets, and all hormones except for gastrin decreased significantly with weight loss (Table 3). Basal glucagon tended to decrease to a lesser extent following the CRHP diet when compared with the CD diet (16 [−0.1, 34]%, *P* = 0.05), whereas glucagon net AUC was reduced significantly more after the CRHP diet (−2.0 [−3.4, −0.6] mmol/L ×240 min, *P* < 0.01, Figure 3). Moreover, GIP total AUC, but not net AUC, was higher on the CRHP than on the CD diet (14 [3, 27]%, *P* = 0.01), and a tendency for higher peak was found (Supplementary Table 2). No differences between diets were found in the OGTT response of GLP-1, CCK, and gastrin (Figures 3, 4). Nonetheless, peaks and total AUCs for these hormones except for gastrin decreased with weight loss which only minutely affected net AUCs and time to peak/nadir.

**Table 3:** Basal concentrations and responses to an oral glucose tolerance test of glucagon, GLP-1, GIP, CCK, and gastrin at baseline and after matched ~6% weight loss by a CD or a CRHP diet in individuals with T2D and overweight or obesity.

	**CD diet**, ***n*** = **33**		**CRHP diet**, ***n*** = **34**		**Between diets**	
	**Baseline**	**Change^a^**	**Baseline**	**Change^a^**	**Difference^b^**	***P* value**
Plasma glucagon						
Basal glucagon, pmol/L	17.6 (15.7, 19.7)	−30 (−37, −22)^‡^	17.8 (16.1, 19.6)	−19 (−28, −9)^‡^	16 (−0.1, 34)	0.05
AUC, pmol/L x 240 min	14.2 (12.7, 15.8)	−30 (−39, −20)^‡^	13.7 (12.6, 14.9)	−27 (−33, −20)^‡^	3 (−10, 19)	0.65
Plasma GLP−1
Basal GLP−1, pmol/L	6.6 (5.1, 8.7)	−65 (−75, −51)^‡^	5.7 (4.2, 7.7)	−42 (−62, −14)^‡^	48 (−4, 128)	0.08
AUC, pmol/L x 240 min	13.3 (11.2, 15.8)	−30 (−43, −14)^‡^	10.8 (9.4, 12.5)	−20 (−32, −7)^†^	3 (−18, 30)	0.79
Plasma GIP
Basal GIP, pmol/L	9.9 (8.0, 12.4)	−59 (−69, −45)^‡^	7.5 (6.0, 9.5)	−43 (−58, −23)^‡^	22 (−17, 78)	0.31
AUC, pmol/L x 240 min	28.7 (25.7, 32.0)	−17 (−25, −9)^‡^	23.4 (21.2, 25.9)	−4 (−10, 1)	14 (3, 27)	0.01
Plasma CCK^c^
Basal CCK, pmol/L	1.1 (0.8, 1.7)	−40 (−55, −20)^‡^	1.1 (0.7, 1.6)	−30 (−49, −4)^*^	14 (−24, 72)	0.52
AUC, pmol/L x 240 min	1.4 (1.0, 1.8)	−22 (−30, −12)^‡^	1.3 (0.9, 1.8)	−12 (−21, −2)^*^	12 (−4, 31)	0.14
Plasma gastrin^c^
Basal gastrin, pmol/L	13.2 (10.0, 17.4)	−11 (−22, 2)	10.3 (8.1, 13.2)	−11 (−21, 0.3)^*^	−4 (−19, 13)	0.61
AUC, pmol/L x 240 min	14.3 (11.5, 17.8)	−8 (−15, −2)^*^	11.3 (9.4, 13.6)	−2 (−8, 4)	3 (−5, 12)	0.43

*Data are presented as mean (95% CI) following log-transformation. Between-diet differences are estimated marginal means (CRHP vs. CD) derived from constrained linear mixed models with inherent baseline adjustment using all available data.*

a *Relative change (%) from baseline.*

b *Relative difference (%) between diets.*

c *Total analyzed n = 66 (CD 32 and CRHP 34). Missing data due to insufficient plasma required for analysis.*

* *P <0.05,*

† *P <0.01, and*

‡ *P <0.001 vs. baseline.*

*CCK, cholecystokinin; CD, conventional diabetes; CRHP, carbohydrate-reduced high-protein; GIP, gastric inhibitory polypeptide; GLP-1, glucagon-like peptide-1*.

### Proinsulin-like molecules

When compared with the CD diet, the CRHP diet did not reduce absolute concentrations of intact, 32,33 split, or total proinsulin; or total proinsulin in relation to insulin or C-peptide (Table 4). However, all variables were reduced significantly and similarly after weight loss (by 28–51%, *P* < 0.001 for all). Participants benefitted robustly as all but one experienced a reduction in the total proinsulin/C-peptide ratio.

**Table 4:** Basal concentrations of proinsulin-like molecules and markers of inflammation at baseline and after matched ~6% weight loss by a CD or a CRHP diet in individuals with T2D and overweight or obesity.

	**CD diet**, ***n*** = **33**		**CRHP diet**, ***n*** = **34**		**Between diets**	
	**Baseline**	**Change^a^**	**Baseline**	**Change^a^**	**Difference^b^**	***P* value**
Proinsulin^c^						
Intact proinsulin, pmol/L	9.5 (7.6, 12.0)	−45 (−54, −35)^‡^	9.4 (7.8, 11.3)	−43 (−50, −36)^‡^	1 (−16, 22)	0.88
Split 32,33 proinsulin, pmol/L	14.5 (11.3, 18.6)	−49 (−58, −38)^‡^	12.8 (10.2, 16.2)	−51 (−58, −43)^‡^	−9 (−27, 15)	0.43
Total proinsulin^d^, pmol/L	24.2 (19.1, 30.7)	−47 (−56, −37)^‡^	23.0 (19.1, 27.6)	−48 (−55, −40)^‡^	−4 (−22, 17)	0.66
Total proinsulin / insulin	0.19 (0.17, 0.23)	−28 (−36, −19)^‡^	0.20 (0.17, 0.23)	−29 (−39, −19)^‡^	−3 (−17, 13)	0.69
Total proinsulin / C-peptide	0.019 (0.016, 0.023)	−38 (−45, −29)^‡^	0.018 (0.016, 0.021)	−41 (−48, −34)^‡^	−9 (−21, 4)	0.18
Inflammation markers^d^						
CRP, ng/mL	1.4 (0.9, 2.0)	−33 (−50, −10)^†^	1.5 (0.9, 2.4)	−14 (−45, 33)	43 (−10, 127)	0.14
TNF-α, pg/mL	2.0 (1.8, 2.2)	8 (0.8, 17)^†^	2.0 (1.8, 2.2)	1 (−3, 6)	−7 (−14, 1)	0.08
IL-6, pg/mL	1.1 (1.0, 1.3)	0.1 (−10, 12)	1.2 (1.0, 1.4)	11 (−8, 35)	12 (−9, 38)	0.29
IL-8, pg/mL	7.4 (6.6, 8.4)	−1 (−13, 11)	6.8 (6.1, 7.5)	−5 (−11, 2)	−6 (−17, 6)	0.28

*Data are presented as mean (95% CI) following log-transformation. Between-diet differences are estimated marginal means (CRHP vs. CD) derived from constrained linear mixed models with inherent baseline adjustment using all available data.*

a *Relative change (%) from baseline.*

b *Relative difference (%) between diets.*

c *Total analyzed n = 64 (CD 30 and CRHP 34). Missing data due to hemolyzed (n = 1) or insufficient serum required for analysis (n = 2).*

d *Total analyzed n = 52 (CD 25 and CRHP 27) for CRP and n = 66 (CD 33 and CRHP 33) for IL-6. Missing data due to measurements under (n = 2) and above (n = 3) the detection range, and intra-assay CV >20% exclusion (n = 11).*

† *P <0.01 and*

‡ *P <0.001 vs. baseline.*

*Total proinsulin, sum of intact and split 32,33 proinsulin; CD, conventional diabetes; CRHP, carbohydrate-reduced high-protein; CRP, C-reactive protein; TNF-α, tumor necrosis factor α; IL, interleukin*.

### Low-grade inflammation

None of the included markers of inflammation differed between groups after the diet interventions, and few changed with weight loss, except for CRP and TNF-α which were reduced and increased, respectively, on the CD diet (Table 4). Notably, the data included much variation and outliers for TNF-α and IL-6 (Supplementary Figure 1), but the results remained robust after excluding these data points (not shown).

### Adverse events

No serious adverse events occurred during the study. Reported adverse events were generally mild and not statistically different between groups. Nevertheless, one participant (CRHP) experienced episodes of transient excessive sweating and increased plasma creatinine concentration but no underlying medical cause was identified. Also, more time of the day was spent with plasma glucose below 3.9 mmol/L (from 7-day continuous glucose monitoring) while on the CRHP diet (21) which, however, did not correspond to symptoms of hypoglycemia. Most adverse events experienced by the participants were gastrointestinal with symptoms of constipation being the most predominant (CD 5, CRHP 8); all but one was easily remedied by sufficient fluid intake and laxatives. Few episodes of diarrhea (CD 2, CRHP 2), dizziness (CD 1, CRHP 2), and feelings of increased tiredness or lack of energy (CD 0, CRHP 2) occurred.

## Discussion

We hypothesized that a clinically relevant weight loss induced by a CRHP diet would ameliorate β-cell dysfunction, impaired glucose tolerance, and stress on the β-cells to a greater extent than a matched weight loss induced by a CD diet in individuals with T2D and overweight or obesity. Instead, we found no improvements in these measures when consuming a CRHP diet relative to a CD diet, but rather a small increase in the secretion of insulin and C-peptide during an oral glucose challenge, despite similar decrements in glucose. Nevertheless, weight loss, as expected, was highly effective in improving β-cell function, insulin sensitivity, and proinsulin processing independently of diet composition.

Weight loss has consistently been shown to be a key component of effective treatment in T2D; indeed, a 15 kg reduction has been shown to almost eliminate hyperglycemia and result in the remission of T2D (11). In fact, this amount of weight loss and/or severe energy restriction may lead to the normalization of β-cell function and hepatic insulin sensitivity to levels typical of individuals without T2D (33, 34). Accordingly, in the present study, we found that the modest 6% weight loss significantly improved β-cell function and responsiveness to glucose and whole-body insulin sensitivity in response to an OGTT, independent of diet composition. We have previously demonstrated—using diets of similar composition and for the same duration—that a moderate substitution of carbohydrate with protein and fat improves β-cell responsiveness to glucose in response to CHRP vs. CD meals (19), but this could have been because of the acute changes specific to the different composition of the CRHP and CD meals (22). This is likely, given that we did not confirm these observations in this study, in response to the same oral challenge (OGTT). It is also likely that the effects of concurrent weight loss in the present study far outweighed and masked any independent effects of macronutrient composition. However, the use of an OGTT may be limited when evaluating carbohydrate restriction as the response in healthy individuals was found to depend on the carbohydrate content of the preceding dinner meal (32).

The β-cell sensitivity to glucose and to incretin hormones seems to improve from short-term amelioration of hyperglycemia by intensive insulin therapy (5, 6), which possibly relieves β-cells from the toxic byproducts of increased insulin production in response to hyperglycemia and the deteriorating effect of glucotoxicity (35). In T2D, stressed β-cells increase the proportion of secreted proinsulin and proinsulin conversion intermediates that remain inactive precursors of insulin, altogether suggestive of impaired intracellular processing of proinsulin to insulin (9, 36). Accordingly, β-cell rest induced by overnight administration of somatostatin reduces proinsulin secretion and improves β-cell function (7). We recently demonstrated that the release of proinsulin-like molecules was decreased after 6 weeks of a weight-maintaining CRHP diet when compared with a CD diet, which we suggested was the result of decreased demand on the β-cells (19), caused by reductions in postprandial hyperglycemia occurring after the CRHP meals (18, 22). We could not replicate this finding in the present study, as there was no difference in basal proinsulin secretion between diets despite the improved glycemic control including a considerably larger reduction in mean diurnal glucose, by 0.8 mmol/L (~50%), after the CRHP diet compared with the CD diet [discussed in Thomsen et al. (21)]. In fact, in response to the OGTT, we found minute increments in ISR and C-peptide total AUCs and peak values after the CRHP diet compared with the CD diet (by ~10%, *P* < 0.05 for both); this was despite identical decreases in glucose responses to the OGTT. The clinical relevance of this finding remains uncertain, but could potentially translate into increased demand on the β-cells when transitioning from a carbohydrate-restricted diet to a carbohydrate-rich diet. Nevertheless, weight loss in the present study, induced by either diet, ameliorated inappropriate proinsulin secretion efficiently, consistent with what others have found (37).

GLP-1 and GIP have well-known insulinotropic properties during glucose stimulation, which account for most of the incretin effect (38), but other secretagogues including glucagon through intra-islet crosstalk (39) and circulating CCK and gastrin are also recognized for contributing to insulin secretion (40). In the present study, only GIP total AUC increased significantly after the CRHP diet compared with the CD diet, which may explain the increase in insulin secretion although the action of GIP in T2D is usually impaired (41). Basal glucagon concentration tended to be reduced to a smaller extent by the CRHP diet than the CD diet, but was suppressed similarly during the OGTT, resulting in a significant reduction in net AUC. Several studies have found that fasting hyperglucagonemia is associated with fasting hyperglycemia through increased hepatic glucose production (39, 42), but the higher glucagon concentration in the CRHP group was not accompanied by a concomitant increase in basal glucose concentration in the present study. We have previously demonstrated that a CRHP meal elicits an increased postprandial glucagon response (because of its higher protein content) even after 4 h (19, 22) and, thus, the observed difference may simply reflect a higher postprandial glucagon secretion from the preceding CRHP dinner, maintained overnight, although some studies suggest that this effect dissipates after an overnight fast (43, 44).

In the present study, the triglyceride total AUC was reduced more by the CRHP diet than the CD diet. This was likely because of the greater reduction in basal triglyceride concentration, maintained during the OGTT. We have previously speculated that decreased *de novo* lipogenesis following each CRHP meal accounted for the observed intrahepatic fat mobilization and improved dyslipidemia (21). Lipotoxicity may affect the β-cells (45), and several studies have found intrapancreatic fat accumulation to be inversely associated with β-cell function in T2D (46). Accordingly, the normalization of β-cell function by short-term severe energy restriction and weight loss was found to depend on the reduction in pancreatic fat in one study (33), which was partly corroborated by the present study as participants in both groups experienced a decrease in intrapancreatic fat [previously published in Thomsen et al. (21)] and an increase in β-cell function. Nonetheless, the role of macronutrient composition is still unclear (18, 21).

Chronic low-grade inflammation is involved in β-cell dysfunction in T2D (47), presumably due to increased proinflammatory adipokine release from enlarged and dysfunctional adipose tissue depots (48). Accordingly, diet-induced weight loss attenuates markers of systemic inflammation in individuals with obesity and T2D (49) and in individuals with obesity irrespectively of dietary carbohydrate content (50). In contrast, individuals with T2D may benefit more from weight loss induced by a severely carbohydrate-restricted diet (20E% vs. 55–60E% from carbohydrates) (51). At odds with these observations, the systemic inflammation (CRP, IL-6, IL-8, and TNF-α) was not ameliorated by the moderate carbohydrate restriction in the present study or by weight loss itself, which suggests that the resolution of inflammation is not an important factor in the metabolic improvement after modest weight loss. These findings corroborated those of a weight-maintaining CRHP diet in a similar study design reported by us previously (52).

The primary strength of this study was the highly controlled setting with full provision of the experimental diets, which we believe is necessary to minimize poor dietary adherence in assessing different eating patterns. Adherence to the present diets was confirmed by measuring 24-h urea excretion in urine [i.e., a surrogate marker of protein intake, published in Thomsen et al. (21)]. Moreover, conclusions on dietary regimens are often confounded by competing lifestyle interventions, e.g., exercise or weight loss unequally distributed between groups or with significant interindividual variation, which we minimized by inducing a matched weight loss between groups and reinforcing and confirming the maintenance of habitual physical activity throughout the study period (21). Medication use was also unchanged. Nevertheless, our study has several limitations, namely, unblinded study design, lack of objective assessment of physical activity, imbalance in sex and DPP-4 inhibitor use between groups, and issues of multiple comparisons that require our results to be interpreted with caution. In addition, we did not properly assess gastric emptying which may increase following a CRHP meal (19) and thereby could influence the metabolic response to the OGTT. However, as the time of peak plasma glucose did not differ between diets, differences between groups in gastric emptying of ingested glucose were likely negligible. When substituting carbohydrates with protein and fat, we allowed other dietary components to vary naturally, reflecting the real foods used. As such, the CRHP diet had more monounsaturated fat and less dietary fiber than the CD diet, and these nutrients may have affected our outcomes independent of carbohydrate restriction. Finally, the duration of our intervention was 6 weeks which may not be sufficient to allow for all metabolic changes to manifest and thus, may not accurately reflect what could be expected in people with T2D undergoing dietary modification for a longer period of time. This is particularly true for the primary outcome of this study, HbA_1c_, which may not have reached a new steady state within 6 weeks, as discussed previously (21).

In conclusion, a modest ~6% weight loss induced after 6 weeks on a diet moderately restricted in carbohydrates and enriched in protein and fat did not improve markers of β-cell function, insulin sensitivity, or proinsulin processing to a greater extent than the same amount of weight loss induced after 6 weeks on a conventional carbohydrate-rich diet despite improved glycemic control. Weight reduction in itself was very efficient in ameliorating metabolic dysfunction, independently of dietary macronutrient composition. These results reinforce the key role of weight loss in the management of T2D.

## Data availability statement

The raw data supporting the conclusions of this article will be made available by the authors, without undue reservation.

## Ethics statement

The studies involving human participants were reviewed and approved by Health Ethics Committee of Copenhagen. The patients/participants provided their written informed consent to participate in this study.

## Author contributions

MT was involved in study planning and execution, including the collection and analysis of data, and the writing of the manuscript. MS and AS assisted with study planning and data interpretation. AA, JH, SM, and JF contributed to the study conception and design as well as the data production and interpretation. TL and FM contributed to the study design, supervised the food preparation and distribution, and assisted in data interpretation and manuscript writing. MF, BH, and JR contributed to the production and interpretation of data. SH and TK were responsible for conceptualizing the study, obtaining funding, supervising the study, and assisting with data interpretation. As guarantors of this paper, MT, SH, and TK take responsibility for the data integrity and accuracy of the data analysis. All authors contributed to the critical revision of the manuscript and gave their approval to the final version for publication.

## Funding

This study was funded by grants from Arla Foods amba, The Danish Dairy Research Foundation, and Copenhagen University Hospital Bispebjerg Frederiksberg. These study sponsors were not involved in the study's design, data collection, analysis, interpretation, or report writing, and they did not place any limits on the publication of the report.

## Conflict of interest

Author AA is a member of the advisory board/consultant for Gelesis (USA), Groupe Éthique et Santé (France), and Weight Watchers (USA) as well as co-owner of the University of Copenhagen spin-off Flax-Slim ApS and co-inventor on a pending provisional patent application for the use of biomarkers to predict responses to weight loss diets and other related patents and patent applications that are all owned by the University of Copenhagen in accordance with Danish law. He has also co-authored several diets and cookery books, including books on personalized diets. He is not a proponent of any particular diet (e.g., veganism, Atkins diet, gluten-free diet, high animal protein diet, or dietary supplements). Author JH is a member of advisory boards for Novo Nordisk. Author TL is an advisor for the “Sense” diet program. The remaining authors declare that the research was conducted in the absence of any commercial or financial relationships that could be construed as a potential conflict of interest.

## Publisher's note

All claims expressed in this article are solely those of the authors and do not necessarily represent those of their affiliated organizations, or those of the publisher, the editors and the reviewers. Any product that may be evaluated in this article, or claim that may be made by its manufacturer, is not guaranteed or endorsed by the publisher.
