# Supplementary material for: Weight loss improves β-cell function independently of dietary carbohydrate restriction in people with type 2 diabetes: A 6-week randomized controlled trial
Source: Front Nutr. 2022 Aug 19;9:933118. doi: 10.3389/fnut.2022.933118 (PMC9437620; doi:10.3389/fnut.2022.933118)
Supplement: Supplementary file 1 [file Table_1.PDF]

Supplementary Material

Supplementary Table 1. Composition of the CD and CRHP diets (standardized to an energy intake of 2,390 kcal/day or 10,000 kJ/day)

Diet composition (7-day menu)	CD diet	CRHP diet
Total carbohydrate, E%	50.0 (±0.2)*	30.0 (±0.2)*
Simple carbohydrate, E%	11.4 (±2.7)§	6.1 (±2.8)§
Fiber, g	48 (±4)‡	36 (±6)‡
Total protein, E%	17.0 (±0.2)*	30.0 (±0.1)*
Dairy protein, E%	3.0 (±1.0)*	10.7 (±1.8)*
Animal protein, E%	8.9 (±0.7)*	23.0 (±1.0)*
Total fat, E%	33.0 (±0.1)*	40.0 (±0.2)*
Saturated fatty acid, E%	9.9 (±1.8)*	11.5 (±3.0)*
Monounsaturated fatty acid, E%	13.1 (±0.7)†	18.2 (±3.1)†
Polyunsaturated fatty acid, E%	6.5 (±1.2)*	7.0 (±1.2)†
Omega-3 fatty acid, E%	0.8 (±0.3)§	0.9 (±0.5)§
Trans fat, g	0.9 (±0.5)§	0.7 (±0.5)§
Cholesterol, mg	375 (±84)§	438 (±198)§
Calcium, mg	878 (±205)§	1,605 (±512)§
Sodium, mg	2,222 (±812)§	2,169 (±707)§
Potassium, mg	3,135 (±512)§	3,165 (±630)§

Data are presented as mean (±SD) and were generated using Dankost Pro software. Validity (defined as percentage of registered food items specifying this dietary component, weighted relatively to their energy contribution) was suboptimal for some dietary data: * ≥95-100; † ≥85-95; ‡ ≥70-85; § ≥45-70. Energy per cent (E%) is calculated by assuming 4, 4, and 9 kcal of energy per gram of carbohydrate, protein, and fat, respectively. CD, conventional diabetes; CRHP, carbohydrate-reduced high-protein
