# Supplementary material for: Weight loss improves β-cell function independently of dietary carbohydrate restriction in people with type 2 diabetes: A 6-week randomized controlled trial
Source: Front Nutr. 2022 Aug 19;9:933118. doi: 10.3389/fnut.2022.933118 (PMC9437620; doi:10.3389/fnut.2022.933118)
Supplement: Supplementary file 2 [file Table_2.PDF]

Supplementary Table 2. Peak/nadir and time to peak/nadir for metabolic markers and gut hormones following an oral glucose tolerance test at baseline and after matched ~6% weight loss by a CD or a CRHP diet in individuals with T2D and overweight or obesity

	CD diet, *n* = 33		CRHP diet, *n* = 34		Between diets	
	Baseline	Change[a]	Baseline	Change[a]	Difference[b]	*P* value
Plasma glucose						
Peak, mmol/L	18.0 (16.7, 19.4)	-17 (-22, -12)[‡]	18.0 (17.1, 19.0)	-13 (-17, -9)[‡]	5 (-2, 12)	0.18
Time to peak, min	94 (87, 101)	7 (-4, 19)	93 (84, 102)	3 (-7, 13)	-5 (-19, 9)	0.48
Serum insulin						
Peak, pmol/L	430 (335, 553)	7 (-4, 18)	403 (319, 509)	20 (9, 32)[‡]	12 (-2, 27)	0.09
Time to peak, min	91 (78, 104)	24 (8, 40)[†]	89 (73, 104)	27 (12, 42)[‡]	2 (-17, 20)	0.84
Serum C-peptide						
Peak, pmol/L	3,190 (2,734, 3,721)	15 (8, 22)[‡]	3,073 (2,682, 3,520)	28 (20, 37)[‡]	11 (3, 20)	<0.01
Time to peak, min	123 (112, 134)	18 (3, 33)[†]	128 (114, 141)	15 (2, 28)[†]	0.4 (-15, 15)	0.96
Insulin secretion rate						
Peak, pmol x kg^{-1} x min^{-1}	9.1 (7.8, 10.6)	20 (13, 29)[‡]	8.7 (7.6, 9.8)	36 (26, 47)[‡]	12 (2, 22)	0.02
Time to peak, min	102 (88, 115)	20 (7, 33)[†]	105 (94, 117)	4 (-7, 16)	-14 (-30, 1)	0.07
Serum triglyceride						
Nadir, mmol/L	1.4 (1.2, 1.6)	-13 (-23, -1)*	1.3 (1.1, 1.6)	-34 (-42, -24)[‡]	-25 (-35, -13)	<0.001
Time to nadir, min	111 (85, 137)	53 (25, 82)[‡]	134 (111, 158)	53 (22, 84)[‡]	18 (-11, 48)	0.22
Serum NEFA						
Nadir, mmol/L	0.16 (0.13, 0.18)	-19 (-29, -7)*	0.14 (0.11, 0.18)	-32 (-45, -15)[‡]	-20 (-36, -0.1)	0.05
Time to nadir, min	165 (152, 177)	11 (-5, 27)	175 (159, 190)	13 (1, 26)[†]	9 (-5, 24)	0.19
Plasma glucagon						
Nadir, pmol/L	11.5 (10.2, 12.9)	-39 (-50, -25)[‡]	10.8 (9.8, 11.9)	-35 (-43, -26)[‡]	4 (-17, 29)	0.76
Time to nadir, min	132 (110, 153)	-34 (-59, -8)*	115 (97, 132)	2 (-25, 29)	19 (-6, 45)	0.14
Plasma GLP-1						
Peak, pmol/L	23.2 (19.5, 27.5)	-19 (-31, -5)*	19.9 (17.6, 22.4)	-12 (-24, 2)*	2 (-16, 25)	0.81
Time to peak, min	56 (41, 72)	-4 (-26, 18)	59 (40, 78)	-11 (-26, 5)	-5 (-22, 12)	0.59
Plasma GIP						
Peak, pmol/L	46.3 (41.3, 51.8)	-21 (-29, -13)[‡]	39.5 (35.4, 44.1)	-9 (-16, -2)[†]	12 (-0.2, 25)	0.06
Time to peak, min	64 (52, 77)	21 (2, 40)[†]	51 (44, 59)	11 (-7, 29)	-20 (-43, 3)	0.09

Supplementary Table 2. *(continued)*

	CD diet, *n* = 33		CRHP diet, *n* = 34		Between diets	
	Baseline	Change[a]	Baseline	Change[a]	Difference[b]	*P* value
Plasma CCK[c]						
Peak, pmol/L	4.5 (3.5, 5.9)	-34 (-45, -21)‡	4.1 (3.2, 5.3)	-17 (-32, 1)*	25 (-3, 62)	0.09
Time to peak, min	18 (9, 28)	7 (-11, 25)	16 (12, 21)	0.1 (-5, 5)	-9 (-24, 6)	0.24
Plasma gastrin[c]						
Peak, pmol/L	20.6 (16.5, 25.7)	-11 (-18, -3)†	16.4 (13.5, 20.0)	-5 (-11, 2)	4 (-5, 15)	0.41
Time to peak, min	20 (8, 32)	17 (-0.4, 34)	32 (17, 46)	-3 (-20, 15)	-11 (-32, 9)	0.27

Data are presented as mean (95% CI) following log-transformation for peak/nadir. Between-diet differences are estimated marginal means (CRHP vs CD) derived from constrained linear mixed models with inherent baseline adjustment using all available data

[a] Absolute change from baseline for time to peak/nadir, and relative change (%) from baseline for peak/nadir

[b] Absolute difference between diets for time to peak/nadir, and relative difference (%) between diets for peak/nadir

[c] Total analyzed *n* = 66 (CD 32 and CRHP 34). Missing data due to insufficient plasma required for analysis

**P* <0.05, †*P* <0.01, and ‡*P* <0.001 vs baseline

AUC, area-under-the-curve; CCK, cholecystokinin; CD, conventional diabetes; CRHP, carbohydrate-reduced high-protein; GIP, gastric inhibitory polypeptide; GLP-1, glucagon-like peptide 1; ISR, insulin secretion rate; NEFA, non-esterified fatty acid
