# Supplementary material for: Weight loss improves β-cell function independently of dietary carbohydrate restriction in people with type 2 diabetes: A 6-week randomized controlled trial
Source: Front Nutr. 2022 Aug 19;9:933118. doi: 10.3389/fnut.2022.933118 (PMC9437620; doi:10.3389/fnut.2022.933118)
Supplement: Supplementary file 3 [file Table_3.PDF]

Supplementary Table 3. Measures of insulin sensitivity and clearance, and β-cell function and responsiveness at baseline and after matched ~6% weight loss by a CD or a CRHP diet in individuals with T2D and overweight or obesity

	CD diet, *n* = 33		CRHP diet, *n* = 34		Between diets	
	Baseline	Change[a]	Baseline	Change[a]	Difference[b]	*P* value
Insulin sensitivity						
ISI_{comp}, L^2 x mg^{-1} x μU^{-1} x 10^{-4}	1.6 (1.4, 1.9)	46 (28, 65)‡	1.7 (1.5, 2.0)	38 (25, 52)‡	-6 (-19, 10)	0.47
Insulin clearance						
MRCi, L x min^{-1}	2.4 (2.2, 2.7)	6 (0.4, 11)*	2.6 (2.3, 2.8)	4 (-0.4, 9)	-1 (-7, 6)	0.77
β-Cell responsiveness						
IGI_{30}, L x kg^{-1} x min^{-1} x 10^{-9}	0.48 (0.41, 0.55)	23 (14, 32)‡	0.48 (0.42, 0.54)	26 (17, 35)‡	3 (-6, 12)	0.58
IGI_{240}, L x kg^{-1} x min^{-1} x 10^{-9}	0.52 (0.43, 0.62)	41 (39, 54)‡	0.50 (0.42, 0.58)	54 (41, 69)‡	8 (-2, 19)	0.15
B_{total}, L x kg^{-1} x min^{-1} x 10^{-9}	0.46 (0.35, 0.61)	63 (39, 91)‡	0.43 (0.35, 0.53)	74 (50, 102)‡	4 (-11, 21)	0.65
β-Cell function						
D_i, L^3 x g^{-2} x min^{-1} x μU^{-1} x 10^{-1}	0.76 (0.59, 0.98)	137 (88, 199)‡	0.75 (0.62, 0.90)	140 (99, 189)‡	0.1 (-19, 24)	0.99

Data are presented as mean (95% CI) following log-transformation. Between-diet differences are estimated marginal means (CRHP vs CD) derived from constrained linear mixed models with inherent baseline adjustment using all available data

[a] Relative change (%) from baseline

[b] Relative difference (%) between diets

**P* <0.05 and ‡*P* <0.001 vs baseline

B_{total}, β-cell responsiveness to glucose; CD, conventional diabetes; CRHP, carbohydrate-reduced high-protein; D_i, disposition index; IGI, insulinogenic index; ISI_{comp}, composite index; MCRi, metabolic clearance rate of insulin
