# Supplementary material for: Weight loss improves β-cell function independently of dietary carbohydrate restriction in people with type 2 diabetes: A 6-week randomized controlled trial
Source: Front Nutr. 2022 Aug 19;9:933118. doi: 10.3389/fnut.2022.933118 (PMC9437620; doi:10.3389/fnut.2022.933118)
Supplement: Supplementary file 4 [file Image_1.PDF]

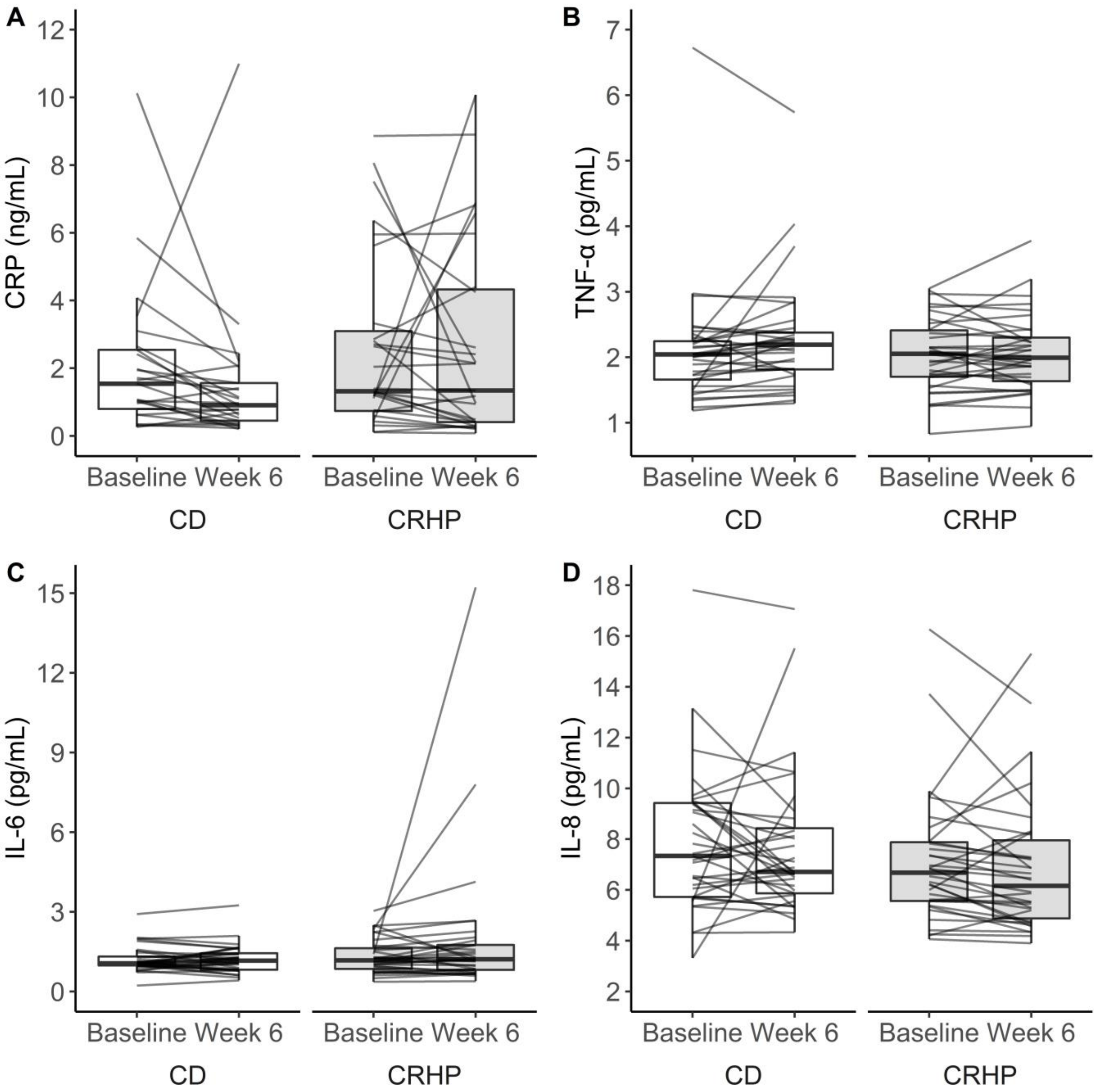

Supplementary Figure 1. Changes in basal plasma concentrations of CRP (**A**), TNF-α (**B**), IL-6 (**C**), and IL-8 (**D**) during 6 weeks of a CD or CRHP diet. Data are presented as median (25th, 75th) with individual changes. No difference between diets were found from linear mixed model analysis: P = 0.19, P = 0.08, P = 0.29, and P = 0.28 for (**A-D)**, respectively
